# Combined repetitive transcranial magnetic stimulation and functional electrical stimulation cycling to improve lower extremity function following incomplete spinal cord injury: Protocol for a pilot randomized controlled trial

**DOI:** 10.1371/journal.pone.0345100

**Published:** 2026-03-19

**Authors:** Fereshteh Ghahremani, Siobhan Schabrun, Sue Peters, Laura Brunton, Janelle Unger

**Affiliations:** 1 Health and Rehabilitation Science, Faculty of Health Sciences, University of Western Ontario, London, Ontario, Canada; 2 Gray Center for Mobility and Activity, Parkwood Institute, St. Joseph’s Health Care Foundation, London, Ontario, Canada; 3 School of Physical Therapy, Faculty of Health Sciences, University of Western Ontario, London, Ontario, Canada; University of Catania: Universita degli Studi di Catania, ITALY

## Abstract

**Background:**

Spinal cord injury (SCI) is a neurological condition that affects thousands of individuals globally each year. Among its many consequences, lower extremity impairments, including reduced walking function, balance deficits, and muscle weakness, significantly impact mobility and quality of life. Various rehabilitation approaches aim to restore function, including neuromodulation strategies, such as functional electrical stimulation (FES) cycling and repetitive transcranial magnetic stimulation (rTMS). While both interventions have shown effectiveness in improving lower extremity function following SCI, they have yet to be performed together to determine the combined effectiveness. This pilot trial will evaluate the feasibility and safety of combining rTMS and FES cycling in people with motor incomplete SCI (iSCI) and provide information to determine a sample size for a full randomized controlled trial. Secondary outcomes will explore the effectiveness of the combined intervention on lower extremity function.

**Methods:**

This is a pilot randomized controlled trial with assessor and participant blinding. Fourteen participants will be randomized to receive either active or sham rTMS prior to FES cycling for a total of 12 sessions over six weeks. Functional outcomes, including gait parameters, muscle strength, and balance tests, will be assessed at baseline, midpoint, post-intervention, and two weeks following treatment cessation and will be assessed both within and between groups to explore trends in efficacy. Feasibility and safety data will be analyzed descriptively.

**Implications:**

This study aims to establish a protocol and sample size for running a full randomized controlled trial on the effectiveness of combining rTMS and FES cycling for lower extremity rehabilitation in iSCI. The protocol is registered with ClinicalTrials.gov (ID: NCT05975606).

## Introduction

Spinal cord injury (SCI) is characterized by damage to the spinal cord and neural pathways within it, including the corticospinal tract (CST), the primary conduit for motor command transmission from the brain to the muscles [1]. People with motor incomplete SCI (iSCI) experience changes in the organization and function of the CST following their injury, including reduced corticomotor excitability [2]. Disruption of the CST following iSCI impairs the transmission of motor commands between the motor cortex and the periphery leading to significant impairments in walking function [3]. To target these impairments, rehabilitation approaches in this population focus on eliciting activity-dependent neuroplasticity in the CST [4,5]. Neuromodulation, the use of central or peripheral nervous system stimulation to alter corticomotor excitability, is one rehabilitation approach shown to facilitate activity-dependent neuroplasticity in the CST and result in improved function for people with iSCI [5].

Functional electrical stimulation (FES), the delivery of electrical currents to stimulate peripheral muscles or nerves during functional activities, is the most commonly used neuromodulation technique in SCI rehabilitation [6,7]. FES cycling facilitates neurorecovery following SCI, with studies reporting improvements in lower extremity muscle mass, bone mineral density, spasticity, strength, and motor output [8–11]. Despite these changes, improvements in overground walking remain limited or variable [11,12]. Taken together, the addition of other interventions with cumulative effects on neuroplasticity in the CST may be needed to improve walking function.

Repetitive transcranial magnetic stimulation (rTMS) is a non-invasive neuromodulation technique used to enhance corticomotor excitability [13], particularly when paired with activity [5]. Implementing rTMS immediately prior to other rehabilitation approaches, including resistance training [14], locomotor training [15–17], and standard care [18], improved lower extremity strength when compared to sham rTMS, but similar to FES, significant improvements in walking function have yet to be observed.

Integrating rTMS with FES cycling represents an innovative approach that could enhance neuroplasticity following iSCI, as pairing two types of neuromodulation is believed to facilitate synaptic pathways through repetitive firing and long-term potentiation [19,20]. It is hypothesized that the combination of rTMS with FES cycling may unlock synergistic neuroplasticity through subsequent cortical excitation and peripheral feedback. There is evidence from paired associative stimulation (PAS) literature indicating pairing peripheral input (through FES currents) with TMS over the motor cortex can achieve Hebbian based increases in corticospinal excitability [21]. In animal models of SCI, simultaneous paired stimulation of the motor cortex and spinal cord over repeated sessions enhanced motor evoked potentials (MEPs) and improved locomotor recovery, confirming multimodal stimulation can reorganize motor networks [22]. Recent evidence in people post-stroke has shown that combining rTMS and FES training resulted in better hand function as measured by the Fugl-Meyer assessment when compared to rTMS or FES training alone [23]. Similarly, a study in people with iSCI found that using rTMS prior to upper extremity FES training resulted in significant improvements on a finger tapping test, pegboard task, the action research arm test, and the modified Sollerman hand function test when compared to a sham control [24]. Overall, these findings support a framework in which rTMS augments descending pathways while FES provides sensory feedback, potentially producing more robust plasticity than either modality alone. Currently, no study has examined the effect of combining rTMS with FES cycling on lower extremity function in people with iSCI.

The primary objectives of this study are to: 1) evaluate the feasibility, acceptability, and safety of a protocol combining rTMS with FES cycling in individuals with iSCI, and 2) provide data for a sample size calculation to inform a full randomized controlled trial. The secondary objective is to explore the effects of combining rTMS with FES cycling on lower extremity function including muscle strength, walking function, and postural sway in quiet standing, to identify trends and inform sample size estimation for future definitive trials. Since the goal of this pilot is to test the feasibility of adding rTMS to an established rehabilitation modality, the design of this trial does not include an rTMS-only arm. This approach is supported by the broader SCI literature, which consistently describes rTMS as most effective when paired with active motor training rather than applied in isolation. Recent reviews note that rTMS is commonly delivered alongside task specific rehabilitation to enhance corticospinal excitability and functional recovery [25,26]. Therefore, all participants will receive FES cycling, and the comparison focuses on real versus sham rTMS to explore early signals of additive benefits.

## Materials and methods

This protocol was prepared according to the 2025 Standard Protocol Items: Recommendations for Interventional Trials statement [27] (S1 File) and the trial will be reported following the Consolidated Standards of Reporting Trials statement for nonpharmacological treatments [28,29] as well as the extension to randomized pilot and feasibility studies [30], the Template for Intervention Description and Replication checklist for rehabilitation intervention and reporting [31], and the Consensus on Exercise Reporting Template [32]. This protocol was registered in ClinicalTrials.gov under the ID: NCT05975606 in August 2023 and is available at https://clinicaltrials.gov/study/NCT05975606.

### Design and setting

**Fig 1:**
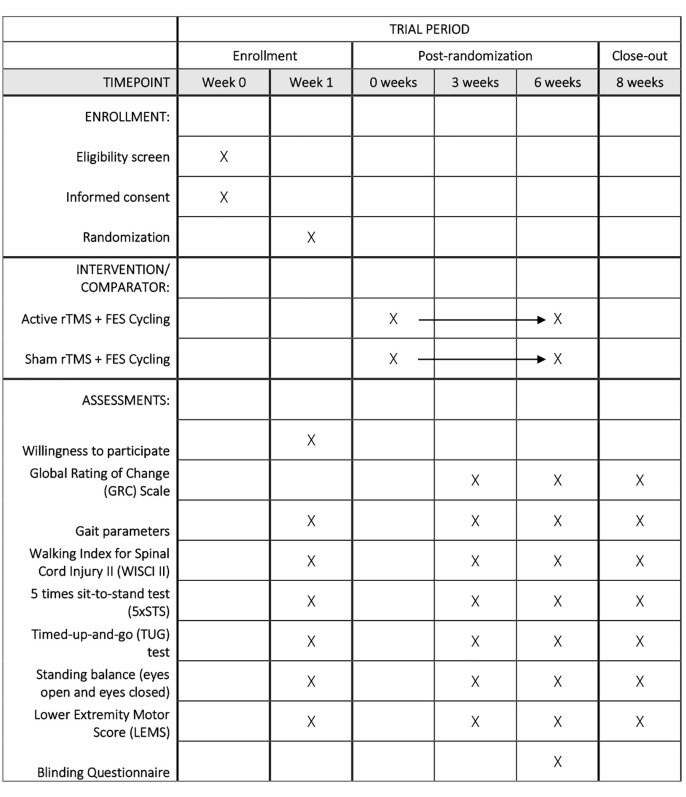
SPIRIT [27] participant timeline.

This study is a double-blind randomized controlled pilot feasibility trial with participant- and assessor-blinding. Participants will be assessed at baseline, mid-point (three weeks post-randomization), after completing the intervention (six weeks post-randomization), and two weeks following intervention completion. As shown in Fig 1, the SPIRIT timeline outlines the full schedule of enrollment, assessments, and intervention sessions across the eight-week study period. The study will take place at the Gray Centre for Mobility and Activity in Parkwood Institute, St. Joseph’s Health Care London, Ontario, Canada and has been approved by the Western University Research Ethics Board (REB #122650) and Lawson Health Research Institute Ethics Board (Approval #R-23-245) in accordance with the Declaration of Helsinki (1964). These boards will be notified on any trial amendments, deviations, or adverse events.

### Participants and recruitment

Participants will be recruited from Parkwood Institute and will first undergo study screening to determine eligibility according to inclusion and exclusion criteria (Table 1). Once determined eligible, all participants will be provided with a letter of information containing all relevant information about study procedures and will provide written informed consent to a study team member prior to initiating any study procedures. Demographic information including age, level and severity of injury, time since injury, sex, and gender will be collected and stored separately from study data. Medication usage will also be documented, and participants will be asked to report any changes in medication over the course of their participation to the research team.

**Table 1:** Inclusion and exclusion criteria.

Inclusion Criteria	Exclusion Criteria
Adults with iSCI (≥18 years of age)	Presence of another neurological or orthopedic disorder affecting lower extremity function
Motor incomplete SCI at any spinal level, either from traumatic or non-traumatic mechanisms	Progressive SCI
Chronic injury (>1 year post-injury)	Contraindication to FES (implanted electronic devices in the body, unhealed fractures, severe contractures in the lower extremity, extreme osteoporosis, extreme osteoarthritis)
Self-reported ability to walk independently for 10 meters (with or without assistive devices)	Contraindications to TMS (epilepsy, history of seizures, severe head trauma, hearing problems, pregnancy, metal in brain or scalp, cochlear implants, implanted neurostimulator, cardiac pacemaker, medication infusion device, spinal or ventricular derivation device)
	Previous experience with rTMS

### Randomization and blinding

Participants will be randomized to either the active rTMS intervention group or the sham rTMS control group using a 1:1 allocation ratio; all participants will receive active FES cycling. Randomization will be blocked by four and stratified by the American Spinal Injury Association Impairment Score (AIS) of C or D. An individual who is not a member of the research team will perform draws for the group assignment. Participant blinding in this protocol will be achieved using a sham rTMS coil, which functions through distribution of the electrical field so there is no focal stimulation over the cortex. As a result, the magnetic field generated is insufficient to induce an electrical response in the underlying tissue. The sham coil is identical in shape, color, and weight to the active coil and produces the same auditory and somatosensory sensations on the scalp associated with active stimulation. The study assessor will also be blinded to group assignment until data analyses are complete. Only the rTMS technician will be aware of group allocation; unblinding will be allowed if an adverse or unexpected event occurs.

### Study procedures

This study will consist of 12 intervention sessions (two per week for six weeks) lasting approximately two hours. Each session will begin with the rTMS procedures immediately followed by FES cycling with changes in health status and side effects monitored throughout each session. Participants will be free to participate in any other exercise or rehabilitation program while in the study.

### rTMS protocol

The rTMS protocol will be conducted using the Magstim Rapid^2^ stimulator (Welcony, Roseville, MN, USA) fitted with a 70 mm figure-of-eight coil, targeting the first dorsal interosseous (FDI) muscle of the right hand. We selected the FDI muscle rather than a muscle in the lower extremity as cortical reorganization following SCI can result in decreased excitability in the lower extremity motor areas [33,34] and the hand area of the motor cortex contributes to lower extremity activation [35]. Additionally, there is substantial evidence for interlimb neural coupling, where activation of hand or arm motor circuits can modulate corticospinal drive to leg muscles. For example, active rhythmic arm cycling has been shown to facilitate MEPs in leg muscles in people with iSCI, indicating functional cross limb interactions [36]. Furthermore, rTMS over the primary motor cortex (M1) has been shown to restore spinal reciprocal inhibition and reduce lower limb spasticity in SCI patients, suggesting that cortical stimulation can influence lower limb function through descending pathways [37]. Consequently, these findings support the rationale for targeting the hand motor cortex to potentially influence lower limb function via interlimb interactions. Targeting the upper extremity also allows for a lower stimulation intensity due to the superficiality of the motor area for the hand, increasing the safety of the protocol. This protocol has been used by other studies evaluating gait in iSCI [15–17]. Several prior gait related rTMS trials in iSCI have also stimulated the FDI representation and still reported meaningful improvements in lower limb function. Nogueira et al. and Benito et al. delivered rTMS over the FDI region and reported enhancements in lower extremity strength, independent walking, and functional gait outcomes [17,18]. These studies demonstrate that stimulation of the hand representation in the motor cortex can facilitate lower limb recovery, supporting the rationale for targeting the FDI in the present protocol. Coil calibration is done to ensure accuracy in TMS delivery over M1 using stereotactic neuronavigation with a curvilinear reconstruction of an averaged brain using Brainsight (Rogue Research, Montreal, QC, Canada). First, single TMS pulses will be used to identify the FDI hotspot and determine the resting motor threshold (RMT). The hotspot is defined as the location within M1 that produces the largest MEPs in the FDI muscle for a given stimulus [38]. The RMT is defined as the minimum stimulation intensity that elicits a peak-to-peak MEP of at least 50μV in at least five consecutive trials [39]. After the hotspot and RMT have been identified, either the active or sham rTMS coil will be attached to the machine by the rTMS technician. rTMS will be administered using either the active or sham coil at 90% of the RMT (20 Hz, 15 trains of 120 stimuli, 28 second intertrain interval), following the protocol used by Benito et al. [18]. The total duration of rTMS delivery will be approximately 17 minutes and include 1800 pulses. The rTMS technician will monitor participants continuously throughout the session.

### FES cycling protocol

An FES bike (Restorative Therapies RT300 – New Hampshire, USA) will be used to deliver sequenced electrical currents to the following muscles bilaterally to perform the cycling motion: gluteus maximii, quadriceps, hamstrings, tibialis anterior, and gastrocnemii. During FES cycling, the stimulation sequence will follow a fixed pattern that is based on pedal position to activate lower limb muscle groups in synchrony with the cycling motion. FES parameters will follow guidance from previous studies in this population with pulse width of 200–400 µs and amplitude of 125–150 mA [12]. The intensity of the electrical currents will be set to a level that elicits a visible muscle contraction but remains tolerable for the participant; intensity will be set during the first intervention session and kept the same in the subsequent sessions unless adjustments are warranted during each intervention session based on participant tolerance. Thereafter, all individual cycling parameters, including any adjustments to stimulation intensity, will be saved under the participant’s profile, ensuring reproducibility across sessions. Duration of FES cycling sessions will be 60 minutes with approximately 15 minutes of equipment set-up and 45 minutes of cycling time and the target cycling speed will be set to 30–35 revolutions per minute (RPM), with resistance ranging from 5–15 N [12]. Although there is a short delay between rTMS and FES cycling, prior studies demonstrate that high-frequency rTMS increases corticospinal excitability for at least 60 minutes after stimulation in individuals with incomplete SCI, suggesting that facilitatory effects on cortical excitability are likely to persist through the FES setup period [40]. The FES bike automatically adjusts based on the participant’s applied force to maintain the target speed. For example, if the participant generates insufficient force, the bike will provide graded assistance to achieve the target RPM; conversely, if the participant exerts greater force, the bike will increase resistance to stabilize the cycling speed. Participants will be instructed to actively contribute to pedaling throughout the session by pushing into the pedals and matching the target cadence with verbal encouragement provide to promote continuous voluntary effort rather than passive movement while emphasizing consistency of speed rather than maximal cadence.

### Outcome measures

Primary outcomes include feasibility, acceptability, and safety of the protocol. To assess feasibility the: 1) time taken to complete the recruitment of all participants, 2) proportion of participants recruited from the total number screened, and 3) blinding success will be collected using a blinding questionnaire where participants will be asked to indicate which study group they believe they were assigned to and rate their confidence in their choice. To measure the acceptability of the protocol: 1) protocol adherence rate (target ≥ 80%), 2) number of dropouts in each of the treatment and sham groups (target ≤ 20%), and 3) the willingness of participants to undergo therapy (in the form of an 11-point scale (0–10) completed during the baseline assessment session, target: at least 7/10 in ≥80% of participants), will be collected. This questionnaire was included based on prior evidence that individuals with SCI are generally interested in participating in physiotherapy based clinical trials but may be less certain when interventions involve electrical stimulation [41]. Safety will be monitored throughout the trial using a standardized adverse event case report form. At each session, participants will be asked about any new or ongoing adverse events, and any event identified will be documented with its onset date, resolution date, detailed description, and severity, classified according to the Common Terminology Criteria for Adverse Events (CTCAE) [42]. Events will also be categorized as serious or non-serious, expected or unexpected, and their relationship to the study procedures will be recorded. For serious adverse events, information on the nature of the event (e.g., hospitalization, life-threatening occurrence, disability) will be captured. The outcome of each event and its impact on study participation will also be documented. All safety data will be reviewed descriptively to inform the feasibility and safety profile of the protocol for a future definitive trial.

Secondary outcome measures include the assessments related to lower extremity function. These outcomes will be collected by a registered physiotherapist who is blinded to group allocation. Outcome measures will be assessed in the same order for all participants. On all assessments after the baseline assessment participants will begin by completing the Global Rating of Change (GRC) scale, which asks participants to rate their perceived improvement in lower extremity function on a scale ranging from −7 (indicating “a very great deal worse”) to +7 (indicating “a very great deal better”) [43]. The 5x sit-to-stand test (5xSTS), the timed-up-and-go (TUG) test, and the tests of standing balance will be conducted using inertial measurement units (IMUs) (Clario – Oregon, USA).

1. Participants will be asked to walk on a 7-metre-long instrumented walkway (ProtoKinetics – Pennsylvania, USA) to collect gait parameters including gait speed, step length, step width, and cadence.
2. The Walking Index for Spinal Cord Injury (WISCI) II will be used to assess walking ability by evaluating the level of physical assistance and use of gait aids. The WISCI II is scored out of 20 with higher scores indicating more independence with walking and has been demonstrated to be a valid and reliable tool for use in the SCI population [44].
3. The 5xSTS test will ask participants to stand up from a seated position on chair with their arms by their sides, sit back with their back touching the chair, and repeat five times. The total time to complete the five repetitions will be recorded. This test has been validated for use in people with iSCI [45].
4. The TUG test will require participants to stand up from a seated position on a chair, walk 3 metres at a comfortable pace, turn 180 degrees, walk back to the chair, turn again, and sit down, with the total duration of time in seconds recorded. This test has been shown to be reliable and valid in the iSCI population [46,47].
5. In the standing balance assessment, participants will stand on a firm surface with their feet shoulder-width apart and their hands on their hips, if able, for 30 seconds with eyes open and then repeated with eyes closed. Mean sway velocity, measured in metres per second, will be measured during this assessment.
6. The Lower Extremity Motor Score (LEMS) will be measured to assess the strength of five key muscle groups in the lower limbs using a six-point grading scale [48]. Each muscle group will be scored separately on both sides of the body, with a maximum of 5 points per muscle. The scores for each lower extremity will be added together for a total out of 50, with higher values indicate better muscle strength.

### Data analysis and management

This is a pilot study designed to inform the development of a protocol for a definitive randomized controlled trial. As this is a pilot trial, the sample size was not determined through a formal power calculation. Instead, the target of 14 participants (7 per group) reflects pragmatic considerations related to recruitment feasibility in individuals with iSCI, a population with known challenges in enrollment [49,50]. Methodological guidance indicates that feasibility focused pilot studies may appropriately include smaller sample sizes when the primary objectives are to assess recruitment, adherence, safety, and protocol procedures rather than to obtain precise estimates of treatment effects [30,51,52]. Pilot trials in SCI population frequently use similarly small samples due to the clinical characteristics and limited pool of eligible participants [49]. Outcomes related to demographics, feasibility, acceptability, and safety will be reported descriptively [53]. For the secondary outcomes, mean scores and mean changes over time will be summarized descriptively to explore preliminary trends in the effectiveness of the intervention. Reasons for any missing values will be categorized and reported, allowing for patterns to be analyzed to evaluate the acceptability of the intervention and the burden of the protocol. Consistent with the exploratory nature of this study, analyses will be interpreted descriptively rather than through formal hypothesis testing, with emphasis placed on patterns of change across time and between groups. Because the study includes repeated measurements, results will be organized by timepoint to reflect within group changes over the study period, and between group differences will be described accordingly. Effect sizes with confidence intervals will be calculated for each group using Cohen’s d to provide estimate of the magnitude of change and to support planning a future definitive trial [30,54]. Given the pilot nature and small sample size, effect sizes will be interpreted cautiously, recognizing their potential instability and risk of overestimation. Lastly, data will be used to inform the sample size calculation for the definitive randomized controlled trial based on the minimum clinically important difference (MCID) in walking speed [55]. In iSCI, the MCID for walking speed is 0.13m/s [56]; power will be set at 80%, alpha at 0.05 and a dropout rate based on the findings of the pilot trial. As this is a single site pilot trial a data safety and monitoring committee (DSMC) is not required.

### Status and timeline

This study is ongoing with participant recruitment and data collection anticipated to be complete by January 2026 and results available in July 2026. Recruitment began on September 7, 2023 and will conclude on Nov 30, 2025. Data will be made available with publication of findings as appropriate.

## Discussion

This pilot RCT aims to establish the preliminary feasibility, safety, and acceptability of pairing rTMS with FES cycling for lower extremity functional improvement in people with iSCI. As completing an exercise based randomized controlled trial in this population is challenging [50], there may be potential limitations in this work. Although a power calculation to determine sample size is not needed, the small number of participants may be heterogeneous, introducing variability [50]. As a result, the precision of the descriptive estimates will be limited, and all findings will need to be interpreted as exploratory. While prior work supports interlimb neural coupling and corticospinal modulation of lower limb following hand area stimulation, direct measures of lower limb MEP levels are not included in the present pilot study and will be important to incorporate in future trials to more directly characterize mechanistic pathways underlying observed functional changes. There can be difficulty recruiting in this population due to the low prevalence relative to other neurological conditions, and although many people with iSCI are willing to participate in clinical trials, fewer are willing to when the research involves stimulation [41]. There may also be retention and adherence limitations, as the protocol does require travel to the site two days per week for six weeks; strategies to support retention include reimbursement for travel and parking costs and offering FES cycling to all participants, as this is typically a paid service for people with SCI living in the community. Although participants will continue their usual rehabilitation routines during the trial, these additional activities will not be formally tracked, which represents a limitation; however, because the study is randomized, any effects of outside therapy are expected to be distributed evenly between groups and therefore unlikely to bias the estimation of the additive effect of rTMS. Future trials building on this feasibility work should incorporate simple activity tracking measures to better characterize external influences on individual responses. The results of this preliminary investigation will lay the groundwork for a larger-scale definitive randomized controlled trial to determine the efficacy of pairing rTMS with FES cycling. Assessing these feasibility outcomes will be crucial prior to running the definitive randomized controlled trial, so that any necessary changes can be made to the protocol. Findings from this pilot trial will be published in academic journals and presented at conferences to inform future research in the field. If pairing rTMS with FES cycling improves walking function for people with iSCI it will be a valuable addition to SCI rehabilitation as the field of neuromodulation continues to grow.

## Supporting information

S1 File SPIRIT checklist. (DOCX)
